# Immobilizing topoisomerase I on a surface plasmon resonance biosensor chip to screen for inhibitors

**DOI:** 10.1186/1423-0127-17-49

**Published:** 2010-06-17

**Authors:** Hsiang-Ping Tsai, Li-Wei Lin, Zhi-Yang Lai, Jui-Yu Wu, Chiao-En Chen, Jaulang Hwang, Chien-Shu Chen, Chun-Mao Lin

**Affiliations:** 1 Graduate Institute of Medical Sciences, Taipei Medical University, Taipei, Taiwan; 2 Department of Biochemistry, School of Medicine, Taipei Medical University, Taipei, Taiwan; 3 Department of Internal Medicine, Taipei Medical University Hospital, Taipei, Taiwan; 4 Institute of Molecular Biology, Academia Sinica, Taipei, Taiwan; 5 School of Pharmacy, PR China Medical University, Taichung, Taiwan

## Abstract

**Background:**

The topoisomerase I (TopI) reaction intermediate consists of an enzyme covalently linked to a nicked DNA molecule, known as a TopI-DNA complex, that can be trapped by inhibitors and results in failure of re-ligation. Attempts at new derivative designs for TopI inhibition are enthusiastically being pursued, and TopI inhibitors were developed for a variety of applications. Surface plasmon resonance (SPR) was recently used in TopI-inhibition studies. However, most such immobilized small molecules or short-sequence nucleotides are used as ligands onto sensor chips, and TopI was used as the analyte that flowed through the sensor chip.

**Methods:**

We established a sensor chip on which the TopI protein is immobilized to evaluate TopI inhibition by SPR. Camptothecin (CPT) targeting the DNA-TopI complex was used as a representative inhibitor to validate this label-free method.

**Results:**

Purified recombinant human TopI was covalently coupled to the sensor chip for the SPR assay. The binding of anti-human (h)TopI antibodies and plasmid pUC19, respectively, to the immobilized hTopI was observed with dose-dependent increases in resonance units (RU) suggesting that the immobilized hTopI retains its DNA-binding activity. Neither CPT nor evodiamine alone in the analyte flowing through the sensor chip showed a significant increase in RU. The combination of pUC19 and TopI inhibitors as the analyte flowing through the sensor chip caused increases in RU. This confirms its reliability for binding kinetic studies of DNA-TopI binders for interaction and for primary screening of TopI inhibitors.

**Conclusions:**

TopI immobilized on the chip retained its bioactivities of DNA binding and catalysis of intermediates of the DNA-TopI complex. This provides DNA-TopI binders for interaction and primary screening with a label-free method. In addition, this biochip can also ensure the reliability of binding kinetic studies of TopI.

## Background

DNA topoisomerases (Tops) regulate the topological state of DNA that is crucial for replication transcription, recombination, and other cellular transactions. Mammalian somatic cells express six Top genes: two TopI (TopI and TopImt), two TopII (TopIIα and β), and two TopIII genes (TopIIIα and β) [1]. TopI produces a single-strand break in DNA, allows relaxation of DNA, and then re-ligates it, thus restoring the DNA double strands. The enzymatic mechanism involves two sequential transesterification reactions [2]. In the cleavage reaction, the active site of tyrosine (Tyr723 in human TopI) acts as a nucleophile. A phenolic oxygen attacks a DNA phosphodiester bond, forming an intermediate in which the 3' end of the broken strand is covalently attached to TopI tyrosine by an O^4^-phosphodiester bond. The re-ligation step consists of transesterification involving a nucleophilic attack by the hydroxyl oxygen at the 5' end of the broken strand. The equilibrium constant of the breakage and closure reactions is close to unity, and the reaction is reversible. Some TopI- and TopII-targeting drugs are reported to stabilize the covalent Top-DNA complex, thereby preventing re-ligation [3]. The TopI reaction intermediate consists of an enzyme covalently linked to a nicked DNA molecule, known as a "cleavable complex". Covalently bound TopI-DNA complexes can be trapped and purified because enzymatic re-ligation is no longer functional. Top inhibitors were developed for antitumor [4], antiviral [5], antibacterial [6], anti-epileptic [7], and immunomodulation [8] applications. Camptothecin (CPT) and its derivatives are representative drugs that target DNA TopI by trapping a covalent intermediate between TopI and DNA, and are the only clinically approved TopI inhibitors for treating cancers. Many derivatives were synthesized, and some of them are in various stages of preclinical and clinical development in recent years. There were more than 150 patents dealing with the modification of the CPT scaffold to obtain derivatives with an improved anticancer activity [9]. Attempts at new derivative designs for TopI inhibition continue to be actively developed. However, several limitations including chemical instability in the blood, susceptibility to multiple drug resistance (MDR), and severe side effects [10] have prompted the discovery of novel TopI inhibitors ahead of CPT.

Surface plasmon resonance (SPR) biosensing is an analytical technique that requires neither radiochemical nor fluorescent labels to provide real-time data on the affinity, specificity, and interaction kinetics of protein interactions [11]. This optical technique detects and quantifies changes in the refractive index in the vicinity of the surface of sensor chips onto which ligands are immobilized. As changes in the refractive index are proportional to changes in the adsorbed mass, the SPR technology allows detection of analytes that interact with the ligands immobilized on the sensor chip [12]. The use of SPR to measure binding parameters for interactions is widely reported. Many applications range from purification [13], epitope mapping, and ligand fishing to identifying small molecules in a screening mode achieved by measuring reaction kinetics (*k*a, *k*d), and binding constants (*K*D). Directly monitoring the binding of low-molecular-mass compounds to immobilized macromolecules has had significant impacts on pharmaceutical discoveries [14].

Methods were developed for TopI-DNA cleavable complex detection to verify TopI inhibitor activity [15,16]. SPR was recently used in TopI-inhibition studies. However, most of those immobilized small molecules or short-sequence nucleotides were used as ligands on sensor chips, and TopI was used as the analyte that flowed through the sensor chip [17,18]. TopI protein preparation is much more complicated than that for DNA, and large quantities of analytes are consumed with large-scale screening using SPR. It would be beneficial to develop an SPR assay with TopI immobilized onto the sensor chip as the ligand to detect TopI-DNA cleavage complexes in response to a variety of analytes.

## Methods

### Reagents and antibodies

Camptothecin (CPT) and evodiamine (EVO) were purchased from Sigma-Aldrich (St. Louis, MO, USA). Enhanced chemiluminescence (ECL) reagents were purchased from PerkinElmer (Waltham, MA, USA). A Plasmid Midiprep Kit was obtained from Promega (Madison, WI, USA). All solvents used in this study were from Merck (Darmstadt, Germany) or Sigma-Aldrich.

### Recombinant human (h)TopI protein expression and purification

Complementary (c)DNAs encoding full-length hTop I were subcloned into the baculoviral expression vectors, pFastBac HTa and pFastBac HTc. The bacmid constructs were prepared using a Bac-to-Bac baculovirus expression system protocol (Invitrogen, Carlsbad, CA, USA). To express and purify the recombinant hTopI, a recombinant baculoviral stock was used to infect 2 × 10^7 ^Sf-9 insect cells per 140-mm plate. Infected cells were cultured at 27°C for 3 days. An Ni-NTA column/imidazole was used for hTopI fractionation [19].

### Western blot analysis

Purified protein samples were resolved by sodium dodecylsulfate polyacrylamide gel electrophoresis (SDS-PAGE) and electrotransferred onto a polyvinylidene difluoride (PVDF) membrane (ImmobilonP, Millipore, Billerica, MA, USA). The membrane was incubated with a primary rabbit antibody against hTopI or γ-H2AX, respectively, at 4°C overnight, and then incubated with a horseradish peroxidase (HRP)-conjugated secondary immunoglobulin G (IgG) antibody; the immunoreactive bands were visualized with PerkinElmer ECL reagents [19].

### Comet assay (single-cell gel electrophoresis)

The comet assay is a widely used method to analyze the consequence of TopI inhibition of DNA integrity, since it enables DNA strand breaks to be detected with high sensitivity at the single-cell level. TopI cleavage complexes are characterized by TopI-concealed single-strand breaks. When TopI is digested by proteasomes, the single-strand breaks collide with replication runoff to form DNA double-strand breaks (DSBs) on the leading strand. To determine the extent of DNA damage in cells, comet assays were performed according to the Trevigen CometAssay™ kit protocol (Trevigen, Gaithersburg, MD, USA) with slight modifications [20]. A2780 cells were treated with 25 μM CPT or EVO for 1 h. The final cell density was about 15,000 cells/mL. The cell suspension (at 50 μL) was then mixed with 500 μL of 0.5% low-melting-point agarose (Invitrogen) at 37°C and subsequently transferred onto glass slides. Slides were then immersed in prechilled lysis buffer (2.5 M NaCl, 100 mM EDTA, 10 mM Tris (pH 10), 10% DMSO, and 1% Triton X-100) for 40 min, followed by electrophoresis in 1× TBE buffer at 1 V/cm for 10 min at room temperature. After electrophoresis, slides were dehydrated in 70% alcohol for 20 min and air-dried. Cells were then stained with SYBR^® ^Green I (Invitrogen) for 5 min. Images were visualized under a fluorescence microscope (IX71, Olympus, Tokyo, Japan) and captured with a CCD camera [21]. On each slide, the nuclei of cells were examined using a fluorescence microscope (Olympus) equipped with an excitation filter of 460~490 nm for detecting DNA migration patterns. Individual tail moments, measured by combining the amount of DNA in the tail with the distance of migration of 50 analyzed cells, were calculated using image analysis software (Comet Assay Software Project, http://www.casp.of.pl/). Tail moment was calculated according to the formula: tail moment = tail DNA% × tail length ([percent of DNA in the tail] × [tail length]). The mean ± S.E. was obtained from at least 50 cells for each treatment group. Statistical analysis was performed using a two-tailed unpaired Student's *t*-test.

### pUC19 plasmid DNA preparation

The pUC19 plasmid was amplified in *Escherichia coli *and purified with the Plasmid Midiprep System (Promega, Madison, WI) following the manufacturer's instructions. The purity was established using the OD 260/280 ratio determined on a NanoDrop ND-1000 spectrophotometer (NanoDrop, Wilmington, DE, USA). Only DNA samples with an OD260/280 ratio of 1.7~1.8 and no degradation on the gel were used for the assays.

### DNA relaxation assay

The inhibitory effect of CPT on supercoiled DNA strand breakage caused by TopI was evaluated. pUC19 plasmid DNA (200 ng) was incubated at 37°C for 30 min in a reaction solution (40 mM Tris-acetate, 100 mM NaCl, 2.5 mM MgCl2, and 0.1 mM EDTA; pH 7.5) in the presence or absence of 2~8 μM of an inhibitor in a final volume of 20 μl. The conversion of the covalently closed circular double-stranded supercoiled DNA to a relaxed form was used to evaluate DNA strand breakage induced by TopI. Samples were loaded onto a 1% agarose gel, and electrophoresis was performed in TAE buffer (40 mM Tris-acetate and 1 mM EDTA). The gel was stained with ethidium bromide (0.5 μg/mL) for 5 min then photographed under transmitted ultraviolet light [22].

### hTopI ligand immobilization on a sensor chip

For immobilization of the recombinant hTopI, hTopI was coupled to the carboxylmethylated dextran surface of a General Layer Medium (GLM) capacity chip (Bio-Rad, Hercules, CA) following the protocol described in the Bio-Rad ProteOn One-Shot Kinetics Kit Instruction Manual with slight modifications [23]. Direct binding experiments were performed on the Bio-Rad ProteOn™ XPR 36 protein interaction array system (Bio-Rad). Briefly, the surface was activated with 0.1 M *N*-hydroxysuccinimide and 0.25 M *N*-ethyl-*N*'-(3-dimethylaminopropyl) carbodiimide at a flow rate of 25 μL/min. hTopI was diluted in 10 mM sodium acetate (pH 7.5) and immobilized at 25°C using a flow rate of 25 μl/min for 288 s (120 μl). Activated carboxylic groups were quenched with an injection of 1 M ethanolamine (pH 8.0). A reference surface was prepared in the same manner excluding hTopI. Immobilization of hTopI was verified by an immediate injection of anti-hTopI antibodies.

### Analyte assay in an SPR sensor chip

Solutions of CPT and/or plasmid DNA pUC19 of known concentrations were prepared in filtered and degassed topo reaction buffer by serial dilutions. All binding experiments were done at 25°C with a constant flow rate of 100 μl/min of Topo reaction buffer (40 mM Tris-acetate (pH 7.5), 2.5 mM MgCl_2_, 100 mM NaCl, and 1 mM EDTA). A DMSO calibration curve was included to correct for refractive index mismatches between the running buffer and inhibitor dilution series. To correct for nonspecific binding and bulk refractive index changes, a blank channel without drugs was used as a control for each experiment. Sensorgrams for all binding interactions were recorded in real time and analyzed after subtracting that from the blank channel. After each measurement, the surface was regenerated with 0.5 M NaCl in 0.05 M NaOH.

### Data processing and analysis

The equilibrium dissociation constants (KD) for evaluating the protein-analyte binding affinity were determined by a steady-state affinity fitting analysis using the results from ProteOn Manager 2.0 (Bio-Rad).

### Computational molecular docking

The X-ray crystal structure of human topoisomerase I-DNA complex [24] was retrieved from the Protein Data Bank http://www.rcsb.org/pdb for docking studies. After addition of hydrogen atoms, the resulting protein-DNA complex structure was used in the docking simulations. The 3-D structure of EVO studied was built and optimized by energy minimization using the MM2 force field and a minimum RMS gradient of 0.05 in the software Chem3D 6.0 (CambridgeSoft, Cambridge, MA). The docking simulations were performed using the GOLD program (version 3.1) [25] on a Silicon Graphics Octane workstation with dual 270 MHz MIPS R12000 processors. The GOLD program utilizes a genetic algorithm (GA) to perform flexible ligand docking simulations. The annealing parameters for hydrogen bonding and Van der Waals interactions were set to 4.0 Å and 2.5 Å, respectively. The GoldScore fitness function was applied for scoring the docking poses using EXTERNAL_ENERGY_WT = 1.375.

## Results

### Purification of hTopI

**Figure 1:**
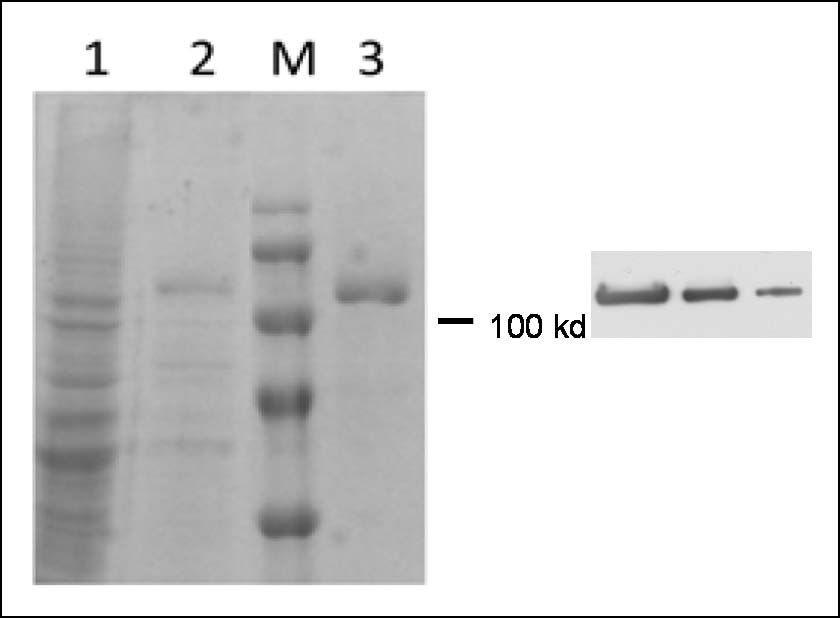
**Purification of recombinant human topoisomerase I (hTopI) obtained using a baculovirus expression system (lane 1, cell lysate; lane 2, partial purified fraction; and lane 3, Ni-NTA column purified protein) (left panel)**. Purified hTopI was further verified by Western blot analyses using serially diluted protein amounts (20, 10, and 5 μg/lane), and probed with rabbit antibodies against hTopI (right).

The recombinant hTopI obtained using the baculovirus expression system was purified. The hTopI expressed by Sf-9 cells was extracted using Triton X-100. Figure 1 shows the different purity levels of the hTopI protein subjected to Ni-column affinity purification. At the final elution from the Ni-column (Fig. 1, lane 3, left panel), purified hTopI was obtained from Sf-9 cells which expressed hTopI. Purified hTopI was further verified by Western blot analyses with serial dilutions (20, 10, and 5 μg/lane) using rabbit antibodies against hTopI (right).

### Inhibition of TopI catalysis by CPT

**Figure 2:**
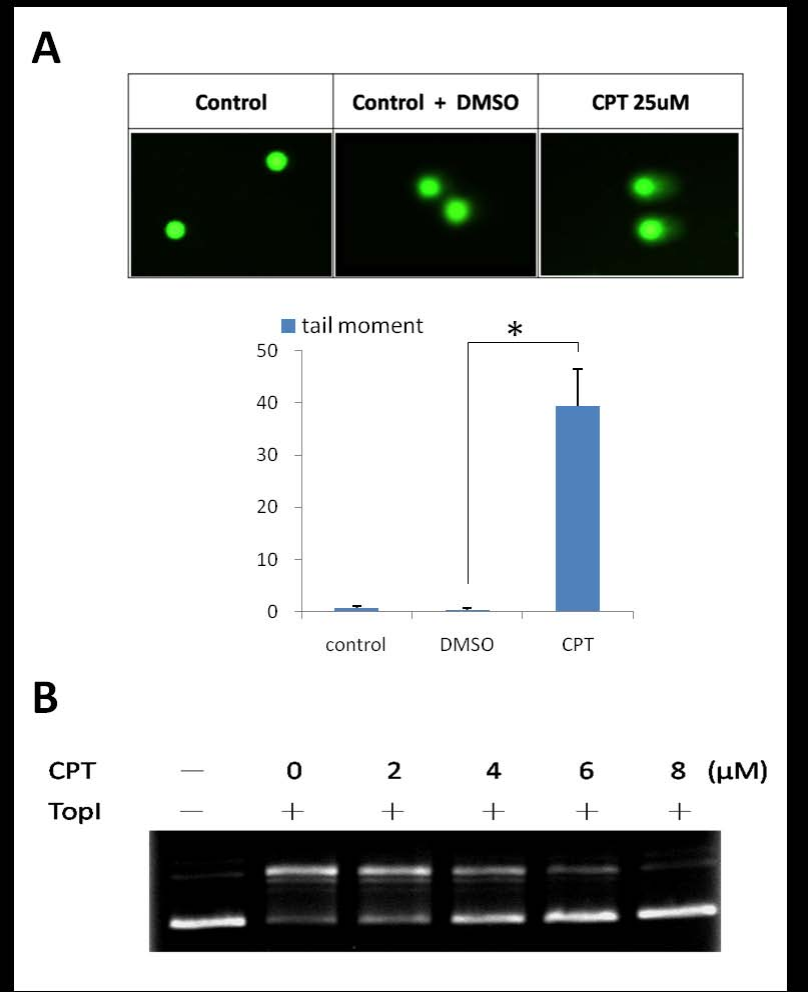
**Inhibitory activity of camptothecin(CPT) on topoisomerase I (TopI)**. (A) CPT-induced DNA damage in A2780 ovarian carcinoma cells. Magnification, ×200. Cells were untreated, treated with DMSO, and CPT (25 μM) for 1 h, and were then analyzed by a neutral comet assay as described in "Materials and Methods." Upper panel, representative images. Lower panel, histogram of the tail moment plotted against each treatment condition. *p *values for comparisons (marked with *) were 0.005 as determined by two-tailed Student's t-test. (B) CPT prevented DNA from recombinant hTop I conversion of supercoiled DNA to relaxed closed circular DNA. pUC19 (0.2 μg) plasmid DNA was incubated at 37°C for 30 min with hTopI in the presence or absence of 2~8 μM of inhibitors.

TopI-DNA cleavage complexes are the key DNA lesion induced by CPT. When single-strand breaks collide with replication runoff, they form DNA DSBs on the leading strand. Figure 2A shows that after treatment with CPT (25 μM) for 1 h, nuclei of control cells presented a compact round area of fluorescence, and no DNA tail was detected. In contrast, treated cells showed DNA tailing, indicating the increased electrophoretic mobility of the DNA fragments, which shows the presence of strand breaks within the nuclear DNA. The addition of CPT to cells enhanced DNA breaks represented by the tailing area calculation (*p *< 0.005, vs. Untreated cells; by Student's *t*-test). An in vitro DNA relaxation assay is often used to measure TopI activity. TopI is known to relax supercoiled plasmid DNA to an open circular form in vitro and in vivo. Here CPT inhibition of supercoiled DNA relaxation in vitro was evaluated. Recombinant hTopI's induction of supercoiled pUC19 plasmid relaxation was used as the assay system, and the results are shown in Figure 2B. Because of their different densities, supercoiled DNA migrated faster on the agarose gel than did relaxed circular DNA shown in the control (Fig. 2B, lanes 1 and 2). CPT treatment inhibited TopI relaxation activity, and a greater amount of uncatalytic supercoiled DNA was retained in a concentration-dependent manner (Fig. 2B, lanes 3~6, 2~8 μM). The results ensure the availability of all materials, including purified recombinant hTopI, the pUC19 plasmid, and CPT, for subsequent assays of TopI catalysis.

### SPR assay of covalent complex formation

**Figure 3:**
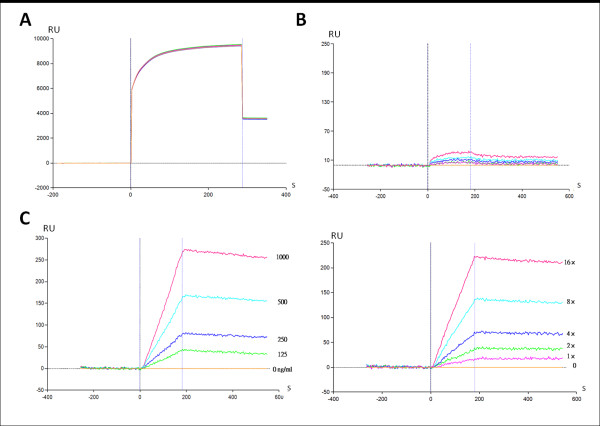
**Surface plasmon resonance sensorgram for the immobilized recombinant human topoisomerase I (hTopI)**. (A) A sensorgram of hTopI immobilized on the General Layer Medium sensor surface. (B) Verification of TopI immobilization using serially diluted polyclonal antibodies against TopI. All curves of lower panel were obtained by subtracting the reference signals from the hTopI-free channel (upper panel). (C) Sensorgram of the interaction between immobilized recombinant hTopI and pUC19 plasmid DNA. Concentrations of DNA were 0~1000 ng/mL. Data are representative of three independent experiments.

**Figure 4:**
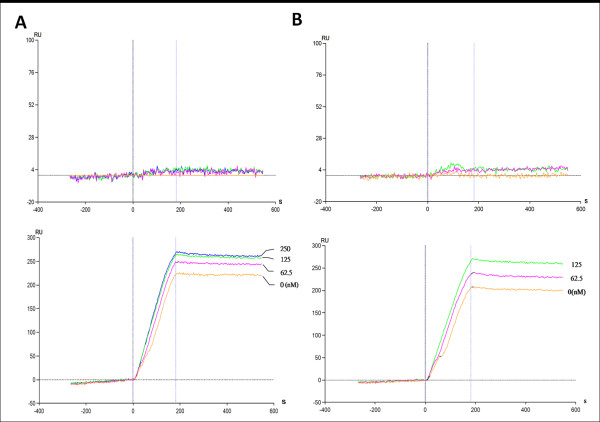
**Surface plasmon resonance sensorgram of the interaction between immobilized topoisomerase I (TopI) and TopI inhibitors**. (A) The interaction of camptothecin (CPT) (0~250 nM) with immobilized recombinant hTopI without plasmid DNA in the analytes (upper panel), and with plasmid DNA (1000 ng/mL) in the analytes (lower). (B) The interaction of evodiamine (EVO) (0~125 nM) with immobilized recombinant hTopI without plasmid DNA in the analytes (upper panel), and with plasmid DNA (1000 ng/mL) in the analytes (lower). Data are representative of three independent experiments.

The SPR assay was used to measure the formation of the DNA-TopI cleavage complex. This assay differs from the gel assay by its high throughput, being in real time and label-free, and directly determining the binding between the analyte and ligand. Recombinant hTopI was covalently coupled to the carboxylmethylated dextran surface of the chip using standard amine-coupling chemistry. The immobilization curves are shown in Figure 3A. The highest level of immobilization was achieved at 4000 RU. The binding of anti-hTopI antibodies to immobilized hTopI was observed in real time after reference subtraction of the response of the hTopI-free control. The response was proportional to the antibody concentration (Fig. 3B, lower panel) while the signals were fairly weak in the hTopI-free channel (upper panel). The pUC19 plasmid was loaded onto the hTopI-immobilized sensor chip, and binding affinities were analyzed. The binding of the pUC19 plasmid to immobilized hTopI was detected by the concentration-dependent increase in RU (Fig. 3C), which suggests that the sensor chip-immobilized hTopI retained its DNA-binding activity. RU values of CPT alone (0~250 nM) in the analyte flowing through the sensor chip remained fairly constant (Fig. 4A, upper panel), which indicates that CPT did not bind to hTopI without DNA. This suggests that the binding of CPT to TopI on the sensor chip was dependent on the DNA content because CPT bound to hTopI at the stage of forming intermediates of the TopI-DNA cleavage complex. To characterize the drug-binding kinetics using the SPR sensor chip, plasmid DNA (1.0 μg/mL) was included in the analyte. The combination of pUC19 plasmid DNA and CPT (0~250 nM) as the analyte was measured flowing through the sensor chip, and the RU increased in a concentration-dependent manner (Fig. 4A, lower) with a KD value of 4.1 × 10^-29 ^(Ka = 9.11 × 10^7^, Kd = 3.74 × 10^-21^) compared to DNA only, according to the ProteOn Manager 2.0 calculation. In the presence of the TopI inhibitor, CPT, re-ligation was impeded; and DNA and TopI were trapped in a covalent cleavage complex. Similar results were obtained with a different TopI inhibitor, EVO, with a KD value of 5.15 × 10^-20 ^(Ka = 7.27 × 10^7^, Kd = 3.74 × 10^-12^) compared to DNA only (Fig. 4B). The interaction caused an increase in the mass of ligand immobilized on the biosensor chip, and was reflected in a rise in RU. The TopII inhibitor, VP-16, did not bind to the TopI-immobilized chip (data not shown).

### EVO binds to TopI and causes DNA damage

**Figure 5:**
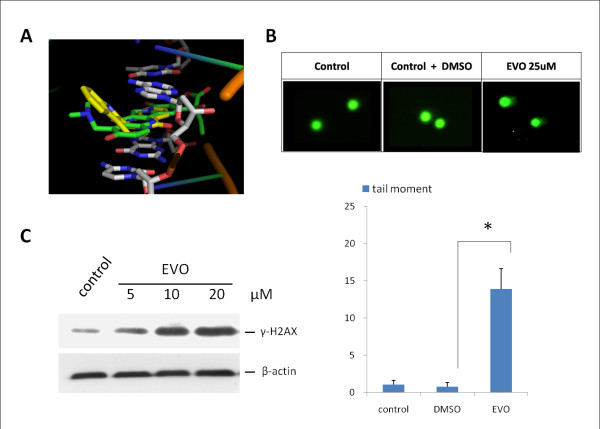
**Evodiamine (EVO) binds to topoisomerase I (TopI) and causes DNA damage. (A) Molecular modeling of camptothecin (green) and EVO (yellow)**. (B) EVO-induced DNA damage in A2780 cells. Magnification, ×200. Cells were untreated, treated with DMSO, and EVO (25 μM) for 1 h, and were then analyzed by a neutral comet assay as described in "Materials and Methods." Upper panel, representative images. Lower panel, histogram of the tail moment plotted against each treatment condition. *p *values for comparisons (marked with *) were 0.005 as determined by two-tailed Student's *t*-test. (C) γ-H2AX levels after EVO treatment in A2780 cells. Cells were treated with 0~20 μM EVO for 6 h. Cell lysates were immunoblotted with antibody against γ-H2AX. β-Actin with constant expression was used as the internal control.

A 3D molecular model was created to evaluate the docking of CPT and EVO to the TopI-DNA cleavable complex. From prior assays, we learned that EVO and CPT are TopI inhibitors which exert similar mechanisms; therefore, they would be expected to dock to the site of the TopI-DNA complex. EVO showed weaker binding (Fig. 5A, yellow, fitness score 67.78) than did CPT (Fig. 5A, green), consistent with the SPR assays (Fig. 4). EVO, which bears a non-planar structure, could not completely intercalate in spaces between DNA bases to form π-π stacking. CPT compactly docked in spaces between DNA bases to form π-π stacking. Results of the structure-based molecular modeling account for the similar bindings of CPT and EVO to the TopI-DNA complex. Figure 5B shows that after treatment with EVO (25 μM) for 1 h, nuclei of control cells presented a compact round area of fluorescence, and no DNA tail was detected. In contrast, treated cells showed DNA tailing, indicating the increased electrophoretic mobility of the DNA fragments, which shows the presence of strand breaks within nuclear DNA. The addition of EVO to cells enhanced DNA breaks represented by the tailing area calculation (*p *< 0.005, vs. untreated cells; by Student's *t*-test). To further verify the DNA-damaging effect on cells, the phosphorylation of histone H2AX (γ-H2AX), a biomarker for DNA DSBs, was detected upon TopI poison treatment. An immunoblot assay was performed to confirm the effect of EVO on γ-H2AX levels, and the result showed that levels of γ-H2AX protein produced by EVO increased in a concentration-dependent manner after 6 h of treatment. The relative level of γ-H2AX after treatment with 0~20 μM EVO increased to > 3-fold versus the control (Fig. 5C). β-Actin with constant expression was used as the internal control.

## Discussion

Small-molecule high-throughput screening of drugs today is mainly designed for those which are dependent upon artificial labels or reporter systems, which can influence the effectiveness due to certain experimental limitations. SPR is known to be a powerful tool for studying biomolecular interactions in a sensitive and label-free detection format. However, label-free methods have been consigned to a supporting role as secondary assays due to throughput and expense constraints. Recent improvements in optical biosensor-based, automated patch clamp and mass spectrometric technologies have enhanced their utility for the primary screening of libraries of small-sized compounds [26]. The major advantages of direct-binding SPR assays compared to other biophysical screening methods are binding kinetic information and very low consumption of the target molecule. Yet SPR assays need reasonably pure and active proteins, as the detection principle is related to detection of the mass measured as a change in the refractive index; there are proteins which are unstable in acidic conditions which are used in the pre-concentration step. This problem can be minimized by mixing the target with the immobilization buffer immediately before injection onto the sensor chip. Antifreeze glycerol is not suitable for use in protein preparation because it causes a severe interference in the refractive index readout. Using DMSO as the antifreeze in the protein preparation significantly reduced this problem.

SPR-based biosensor technologies can directly monitor the binding of small molecules to immobilized macromolecules and thus allow the study of interaction kinetics and the evaluation of binding constants. Immobilization of DNA molecules on sensor chip for drug or protein interactions was successfully established. Immobilization of biotinylated linear or circular DNA on the sensor surface for TopI and topII kinetic assays was performed using an SPR analysis [27-29]. However, determining the binding constant is complicated by multiple binding sites of the target DNA. In addition, in some situations, each binding site has a different intrinsic affinity for binding independently to each binder, which causes a hindrance to determining the affinity constant. Lin et al. provided several modes of determining the binding constant and stoichiometry of DNA-targeting drugs with SPR technology [12]. No previous effort immobilizing Top proteins on sensor chips was able to render binary protein-inhibitor or ternary protein-DNA-inhibitor interaction assays. In addition, there are no plural binding sites for immobilized TopI that make it easier to determine the binding constant. This work is the first demonstration that a Top1-immobilized sensor chip can provide a valid assay of DNA- and inhibitor-binding activities using SPR technology. It also enables a more-precise understanding of the kinetics of TopI reactions.

We preliminarily reported that EVO is a TopI inhibitor that has a variety of potential clinical applications [19]. In the present study, we demonstrated EVO trapping on an established TopI-immobilized sensor chip in the presence of DNA in flow-through analytes. EVO displayed weaker binding activity on the TopI-immobilized sensor chip than CPT in the SPR assay, which is consistent with the results of a DNA-relaxation assay [19]. This result prompted further reliability verification of a new TopI inhibitor using computer-aided molecular modeling, an in vivo comet assay for DNA damage, and the γ-H2AX level, a biomarker for DNA DSBs [2]. The molecular modeling showed that EVO co-docked with the CPT in the binding site of the TopI-DNA-cleavable complex. EVO treatment of A2780 cells caused comet tailing suggesting DNA fragmentation that is a hallmark of Top inhibition. An early response to the induction of DNA DSBs, which can be induced by either TopI or TopII, is phosphorylation of the H2AX at the serine-139 residue, in the conserved C-terminal SQEY motif, forming γ-H2AX [30]. γ-H2AX is predominantly mediated by an ataxia telangiectasia mutation (ATM) through continued phosphorylation proximal to DNA breakage sites which spreads to adjacent areas of chromatin [31]. Increasing γ-H2AX levels in a concentration-dependent manner upon EVO treatment in A2780 cells are consistent with the results of the SPR and comet assays. Taken together with our previous report [19], we concluded that EVO is able to inhibit TopI by formation of the TopI-DNA complex that exerts a similar mechanism as CPT. The results of SPR for EVO were verified using a variety of methods to ensure the reliability of the TopI-immobilized sensor chip. This novel method will be useful for comparing the affinities of various TopI inhibitors and selecting the most suitable candidates for DNA-TopI trapping, as well as facilitating in vitro screening procedures.

## Conclusions

We established and validated a label-free method for evaluating TopI inhibitors using an SPR analysis. TopI immobilized on the chip retained its bioactivities of DNA binding and catalysis of intermediates of the DNA-TopI complex. This provides DNA-TopI binders for interaction and primary screening. In addition, this biochip can also ensure the reliability of binding kinetic studies of TopI.

## Competing interests

The authors declare that they have no competing interests.

## Authors' contributions

HPT carried out the SPR experiments, LWL carried out the Top1 activity assay, ZYL carried out the CPT inhibitory effects on Top1, JYW participated in the study design of SPR, CEC carried out the Top1 expression and purification, JH participated in the study design and coordination, CTC carried out the molecular modeling assay, and CML organized the design of the study and manuscript preparation.

All authors read and approved the final manuscript.
